# Editorial: Biomacromolecule systems for enhanced therapeutic delivery in medical implants

**DOI:** 10.3389/fbioe.2025.1700914

**Published:** 2025-10-13

**Authors:** Joao Henrique Lopes, Nicolas Tabary, Jacobo Hernandez-Montelongo

**Affiliations:** ^1^ Biomaterials and Biointerfaces Lab (BBLab), Department of Chemistry, Division of Fundamental Sciences (IEF), Aeronautics Institute of Technology (ITA), Sao Jose dos Campos, Brazil; ^2^ Université Lille, CNRS, INRAE, Centrale Lille, UMR 8207 —UMET—Unité Matériaux et Transformations, Lille, France; ^3^ Research Center for Bioproducts and Advanced Materials (BioMA), Department of Physical and Mathematical Sciences, Universidad Católica de Temuco, Temuco, Chile

**Keywords:** biomacromolecule, therapeutic delivery, localized delivery, medical implants, drug delievery

The application of biomacromolecule systems in medical implants for the localized release of therapeutic agents is advancing rapidly. This progress is largely fueled by the rising global demand for sophisticated medical devices, particularly in the context of an aging population (Mastnak et al., 2022). Implants such as catheters, stents, and prosthetic joints are indispensable in modern healthcare, serving to replace, support, or enhance biological structures. Yet, surgical implantation is inherently associated with risks, including infection, impaired healing, and complications that may be worsened by immunosuppression linked to conditions such as AIDS, cancer, or diabetes. In this regard, biomacromolecules—large biomolecules including proteins, nucleic acids, and polysaccharides—have shown remarkable potential as carriers for controlled therapeutic delivery. These systems not only reduce postoperative risks but also improve therapeutic outcomes by enabling localized and sustained release of bioactive molecules (Lopes et al., 2022; Rumon et al., 2024). Nevertheless, significant challenges remain. Optimizing biocompatibility, ensuring long-term stability, and fine-tuning release kinetics are essential to fully unlock the clinical potential of these strategies.

Over the past decade, biomacromolecule delivery has evolved from classical polysaccharide and protein carriers into modular and hybrid platforms that merge biological recognition with materials engineering. Emerging modalities include peptide–polymer conjugates and amphiphilic peptides with antimicrobial or pro-regenerative functions; nucleic acid systems (siRNA/miRNA/DNA) condensed by cationic biopolymers for gene-level modulation; and protein–polymer hybrid depots that enable spatiotemporally controlled release of growth factors (Zhao et al., 2025). In parallel, bioinspired coatings and layer-by-layer assemblies on metallic and polymeric implants integrate polysaccharides (e.g., chitosan, hyaluronan), proteins, and catechol-functionalized polymers to improve adhesion, provide on-demand drug elution, and inhibit biofilm formation (Borges et al., 2024). Triggered release mechanisms—responsive to pH, enzymes, redox conditions, or mild external stimuli—are now routine, while translational efforts increasingly focus on hemocompatibility, biofilm prevention, reproducibility, sterilization, and scalable manufacturing. Together, these developments position biomacromolecule systems as versatile interfaces capable of transforming passive devices into active therapeutic platforms.

The field continues to diversify. Hybrid peptide–polymer carriers are being designed for antimicrobial and immunomodulatory applications, coupling intrinsic bioactivity with enhanced stability (Cui et al., 2023). Self-assembling nanosystems—such as peptide amphiphiles, polypeptide micelles, and bioinspired films—are directly integrated onto implant surfaces to achieve contact killing, disrupt quorum sensing, and provide sustained drug release. Nucleic acid payloads (e.g., siRNA, CRISPR guides) are increasingly incorporated into polysaccharide and protein matrices to locally modulate inflammation and fibrosis (Manchanda et al., 2025). These strategies underscore the convergence of biomacromolecule science with surface and polymer engineering, defining a broad and dynamic landscape that frames this Research Topic.

Within this context, the present Research Topic features seven contributions that highlight both experimental and conceptual advances in biomacromolecule-assisted biomedical implants. The original research articles include those by Almuhayawi et al., González et al., and Pulido et al., complemented by reviews from Du et al., Sánchez-Trasviña et al., Calais et al., and Chen et al.. Collectively, these works illustrate strategies spanning drug-eluting coatings, antimicrobial macromolecules, bio–nano interfaces, and metallic implant surface engineering.

Altogether, this Research Topic emphasizes the transformative potential of biomacromolecule-based delivery systems in medical implants. By bridging fundamental science with clinical translation, the contributions presented here point to innovative approaches that may redefine the therapeutic role of implants in modern biomedicine. Beyond the specific examples covered, the scope also embraces peptide–polymer hybrids, self-assembled biointerfaces, and nucleic acid-enabled platforms that are shaping the next-generation of therapeutic implants.
